# Regulation of Focal Adhesion Kinase Signaling by Death‐Associated Protein Kinase 3 in Vascular Smooth Muscle Cells

**DOI:** 10.1002/jcb.70139

**Published:** 2026-11-15

**Authors:** Abdulhameed Al‐Ghabkari, David A. Carlson, Timothy A. J. Haystead, Justin A. MacDonald

**Affiliations:** ^1^ Department of Biochemistry and Molecular Biology Cumming School of Medicine, University of Calgary Calgary Canada; ^2^ Department of Pharmacology and Cancer Biology Duke University Medical Center Durham North Carolina USA

**Keywords:** actin filament, CDC14A, cytoskeleton, DAPK3, FAK, focal adhesion, SMC, ZIPK, zipper‐interacting protein kinase

## Abstract

Death‐associated protein kinase 3 (DAPK3) is a Ser/Thr protein kinase with regulatory involvement in the motility and contractility of vascular smooth muscle cells (VSMC). DAPK3 silencing can induce cytoskeletal remodeling with disassembly of actin stress fiber networks and coincident loss of focal adhesion kinase (FAK)‐pY397 phosphorylation. The link between DAPK3 inhibition and FAK phosphorylation is unknown, and critical interactor(s) and regulator(s) are not yet defined. In this study, we further analyzed the DAPK3‐FAK relationship in VSMCs. As expected, the application of HS38, a selective DAPK inhibitor, to coronary artery vascular smooth muscle cells (CASMCs) suppressed myosin light chain diphosphorylation (LC20, pT18&pS19) and the inhibitory phosphorylation of myosin phosphatase regulators, MYPT1 (pT696) and CPI‐17 (pT38). The suppression of DAPK3 signaling was also associated with a significant decline in FAK‐pY397 phosphorylation (but not pY576). These molecular events were associated with the translocation of cytoplasmic FAK to the nucleus and a loss of CASMC cell migration potential. DAPK3 inhibition with HS38 or silencing with a DAPK3‐siRNA could also attenuate the phosphorylation of other focal adhesion components (p130Cas and paxillin) and the activation of downstream pathways (ERK). Collectively, the data presented herein strongly support a regulatory mechanism for DAPK3 in the indirect control of FAK and focal adhesion signaling in VSMCs.

## 1 Introduction

Focal adhesion kinase (FAK), a cytoplasmic non‐receptor tyrosine kinase, plays a central role in transducing extracellular signals through integrins, enabling mechanotransduction by linking extracellular cues to intracellular signaling pathways that regulate cytoskeletal organization [1, 2, 3, 4]. Not surprisingly, focal adhesions in vascular smooth muscle cells (VSMCs) provide a critical linkage between cytoskeleton and extracellular matrix to enhance membrane rigidity and facilitate the transmission of mechanical tension in these contractile cells [5, 6].

FAK acts as an important signalosome platform to enable complex and dynamic interactions with intracellular structures. These connections govern FAK spatial and temporal activation in coordination with focal adhesion assembly and turnover [3, 4]. Cell adhesion induces FAK activation through autophosphorylation at Y397, creating a docking site for proteins containing SH2 domains [7, 8]. These adapter proteins can recruit multiple signaling partners, thereby integrating FAK into diverse signaling networks. A key event in this cascade is the recruitment of Src, which phosphorylates FAK at Y576 and Y577 within the kinase activation loop [8], thereby enhancing FAK activity and promoting the phosphorylation of downstream focal adhesion components, such as paxillin and p130Cas [1, 9, 10].

FAK is characterized by a FERM domain at the N‐terminus, a central protein kinase domain, a C‐terminal focal‐adhesion targeting (FAT) domain, and an unstructured proline‐rich region between the FAT and the kinase domain [2, 3, 8]. Several studies have demonstrated that FAK can engage with cell cytoskeleton and membrane proteins through the FERM domain [7, 11]. In addition, recent evidence suggests that FAK possesses nuclear localization and export signals (NLS and NES, respectively), suggesting a potential mechanism for FAK nuclear localization, where it impacts transcriptional programming and cell transformation [12, 13]. Although FAK has been extensively characterized, understanding of its spatiotemporal localization, binding interactions, and post‐translational modifications remains incomplete. Indeed, mass spectrometry analyzes have identified several previously uncharacterized phosphorylation sites with undefined mechanistic roles [14]. These phosphorylation events are likely coordinated through a complex interplay between kinases and phosphatases, forming a dynamic regulatory network that is still poorly understood [15, 16].

Recently, a novel signaling relationship was identified for FAK and death‐associated protein kinase 3 (DAPK3, also known as zipper‐interacting protein kinase, ZIPK [17]. The ectopic over‐expression of DAPK3 in human BJ/SV40T fibroblasts was found to alter actin filament bundling and to induce the reorganization of focal adhesions with coincident suppression of pY397‐FAK phosphorylation [18]. In agreement with this study, our group has shown that the suppression of DAPK3 signaling with a small‐molecule inhibitor (i.e., HS38) or siRNA‐mediated knockdown resulted in pronounced alterations in the cytoskeletal architecture of VSMCs with coincident reduction in FAK phosphorylation [19].

DAPK3 has a well‐documented role in VSMC motility and contraction [17, 20]; the kinase also contributes to cell rounding and cell‐matrix detachment of non‐muscle cells [21, 22]. Nonetheless, the precise mechanism whereby DAPK3 regulates FAK phosphorylation and the focal adhesion complex remains undefined. In this study we develop further mechanistic understanding of the FAK and DAPK3 signaling partnership in VSMCs. Central to our experiments is the application of a validated DAPK3 small molecule inhibitor, arylthiopyrazolo[3,4‐d]pyrimidinone (HS38) that competitively inhibits DAPK3 [23, 24]. HS38 provides a unique opportunity to examine DAPK3‐dependent effects on the focal adhesion complex and the actin cytoskeleton in an in vitro setting.

## 2 Materials and Methods

### 2.1 Materials

All chemicals were reagent grade and were purchased from BioRad, Sigma‐Aldrich or VWR. DAPK3‐siRNA (#am4390824) and scrambled siRNA (#amAM4613) were purchased from Ambion/Thermofisher Scientific. HS38 (CAS No. 1030203‐81‐6) was synthesized as previously described [23]. FAK inhibitor 14 (FAK‐IN‐14; CAS No. 4506‐66‐5) was acquired from Sigma‐Aldrich. Antibodies were acquired from: Cell Signaling Technology (anti‐β‐actin, #4967S; anti‐ERK, #9102S; anti‐pERK(pT202/pY204), #4370S; anti‐tubulin, #2144 A; anti‐p130Cas(pY249), #4014 A; anti‐p130Cas(pY410), #4011S; anti‐p130Cas(E1L9H), #13856; and anti‐LC20(pT18/pS19), #3764; anti‐Src(pY527), #2105S), Santa Cruz Biotechnology (anti‐Lamin B, #sc‐6216; anti‐LC20, #sc‐15370), and Abcam (anti‐DAPK3/ZIPK, #ab151956; anti‐FAK, #ab40794; anti‐FAK(pY397), #ab39967; anti‐FAK(pY576), #ab55335; anti‐paxillin(pY118), #ab4833; anti‐SM22α, #ab14106). AlexaFluor488‐Phalloidin, AlexaFluor568 goat anti‐rabbit IgG, AlexaFluor568 goat anti‐mouse IgG and AlexaFluor488 goat anti‐rabbit IgG were purchased from ThermoFisher Scientific.

### 2.2 Vascular Smooth Muscle Cell Culture

Human coronary artery smooth muscle cells (CASMCs, passage 8‐12; CC‐2583, Lonza) were cultured in smooth muscle basal media (SmGM‐2 BulletKit; CC‐3182; Lonza). Cells were confirmed negative for *Mycoplasma* contamination and maintained at 37°C in a humidified atmosphere of 5% CO_2_. Vehicle (DMSO) or DAPK3 inhibitor (HS38, 1 mM stock in DMSO) was diluted in SmGM‐2 media and then added to culture dishes. Typically, CASMCs were treated for 16 h with HS38 or vehicle control before whole cell lysates were collected. Flag‐tagged DAPK3 was transfected into CASMCs using FuGENE 6 transfection reagent (Roche Applied Science) according to the manufacturer's instructions. Briefly, a transfection mixture containing 200 μl Optim‐MEM (Invitrogen), 10 μl FuGENE reagent and 5 μg DNA was added to CASMCs (10 cm^2^ dishes, 70%–80% confluency) and then incubated for 24 h before collecting whole cell lysates. In some cases, DAPK3 knockdown was performed using siRNA transfection. Scrambled‐ and DAPK3‐specific siRNAs were diluted in siRNA transfection medium (sc‐36868, Santa Cruz Biotechnology) to a final concentration of 20 nM and added to CASMCs (2 × 10^5^ cells). After a 6‐h incubation, the transfection medium was replaced with DMEM supplemented with 10% FBS. Cells were harvested 24 h later, and whole cell lysates were collected.

### 2.3 Cell Migration Assay

An in vitro “scratch” assay was used to measure migration of CASMCs. Cells were seeded at 1 × 10^5^ cells/well and then incubated for 48 h. Once the cells reached 70%–80% confluency, vehicle (DMSO) or HS38 was applied to the cells and then incubated for 30 min followed by two washes with PBS. A scratch area was carefully created for cell migration by scraping the cell monolayer with a sterile pipette tip. Detached cells were gently rinsed away with PBS and an image taken by phase‐contrast microscopy. Additional images were acquired after 24 h, and the migratory capacity was assessed by quantifying the area of cell movement into the cell‐free (wound) region.

### 2.4 Subcellular Fractionation

CASMCs were washed into ice‐cold PBS and gently removed from culture dishes. Cells were pelleted in a 1.5 ml Eppendorf by centrifugation (150 g, 1 min), washed twice with PBS, and then resuspended in PBS containing 0.1% (vol/vol) NP‐40 with gentle vortexing. A small fraction was removed to represent the whole cell lysate. The homogenate was centrifuged (700 g, 10 min), and the supernatant was recovered as the cytosolic fraction. The pellet was resuspended in ice‐cold SDS/Laemmli sample buffer (30 min, intermittent vortexing) and then centrifuged (15 000 g, 30 min). The resulting supernatant was recovered as the nuclear fraction.

### 2.5 Western Blotting

Cell lysates were resolved by SDS‐PAGE and transferred to 0.2 μm nitrocellulose membranes in a Tris/glycine transfer buffer containing 10% (vol/vol) methanol. Nonspecific binding sites were blocked with 5% (wt/vol) non‐fat dry milk in Tris‐buffered saline containing 0.1% (vol/vol) Tween‐20 (TBST). Membranes were washed with TBST and incubated overnight at 5°C with primary antibody (typically 1:1,000 dilution in TBST). Membranes were incubated for 1 h with horseradish peroxidase (HRP)‐conjugated secondary antibody (dilution 1:10,000) and developed with enhanced chemiluminescence (ECL) reagent (GE Healthcare). All western blots were visualized with a LAS4000 Imaging Station (GE Healthcare), ensuring that the representative signal was present in the linear range. Quantification was performed by densitometry with ImageQuant TL software (GE Healthcare).

### 2.6 Immunofluorescent Microscopy

CASMCs were plated on coverslips 48 h prior to treatment (vehicle or HS38). Cells were fixed (20 min) in 4% (vol/vol) paraformaldehyde in PBS and then permeabilized with 0.5% (vol/vol) Tween‐20 for 15 min, followed by blocking in 2% (wt/vol) BSA in washing buffer (PBS with 0.2% [vol/vol] Triton X‐100% and 0.05% [vol/vol] Tween‐20) for 30 min. Cells were then incubated at 4°C overnight with primary antibody diluted 1:100 in blocking serum (0.3% BSA, 5% goat serum, 0.3%, Triton X‐100 in PBS, pH 7.4). After washing with PBS, immunoreactivity was detected with goat anti‐rabbit or mouse anti‐rabbit AlexaFluor488‐conjugated secondary antibody. For F‐actin visualization, AlexaFluor488‐phalloidin was diluted 1:40 in 1% (wt/vol) BSA in PBS and then incubated with the cells for 1 h at room temperature. Nuclei were counterstained with 4′,6‐diamidino‐2‐phenylindole (DAPI) for 5 min. Fluorescent microscopy was conducted with an InCell 6000 Imaging System (GE Healthcare). Automatic gain control was used during the image acquisition to maintain a consistent fluorescence signal intensity. The representative images were selected from multiple independent experiments (different cell cultures) and were acquired using identical imaging parameters, including laser power, detector settings and exposure conditions, to allow qualitative comparisons among experimental groups.

### 2.7 Data Analysis

Parametric data are presented as the mean ± S.E.M., with n indicating the number of independent experiments. Statistical significance was assessed using a Student's t‐test or one‐way analysis of variance (ANOVA) followed by Tukey's *post hoc* test, as appropriate. A *p* value of 0.5 was the minimal threshold for statistical significance. All statistical analyzes were performed using GraphPad Prism v6.0.

## 3 Results and Discussion

### 3.1 DAPK3 Signaling is Suppressed in CASMCs With siRNA or HS38 Inhibitor

Immunostaining of CASMCs for endogenous DAPK3 revealed a prominent nuclear pool with an additional diffuse immunoreactive signal observed throughout the cytoplasm (Figure 1A). We found *DAPK3* expression to be effectively silenced in CASMCs with an optimized siRNA treatment protocol (Figure 1B, i). The administration of HS38 did not influence DAPK3 levels (Figure 1B, ii) nor did it significantly impact cell viability ([Supplemental 7]). The link between DAPK3 and myosin regulatory light chain (LC20) diphosphorylation at T18 and S19 has been extensively described in different cell and tissue models [17, 20, 21, 25], and we identified a corresponding decrease in LC20‐2P (pT18/pS19) levels in CASMCs treated with siRNA‐DAPK3 or HS38 inhibitor when compared to the respective control condition (Figure 1C). DAPK3 is also involved in modulating myosin phosphatase (MLCP) activity by phosphorylating the myosin phosphatase targeting subunit (MYPT1) at the inhibitory T696 site and the 17‐kDa C‐kinase‐potentiated inhibitor protein of MLCP (CPI‐17) at the T38 site [17]. We detected attenuated phosphorylation of both MYPT1‐pT696 and CPI‐17‐pT38 in CASMCs when DAPK3 was targeted by either siRNA silencing or pharmacologic inhibition (Figure 1D).

**Figure 1:**
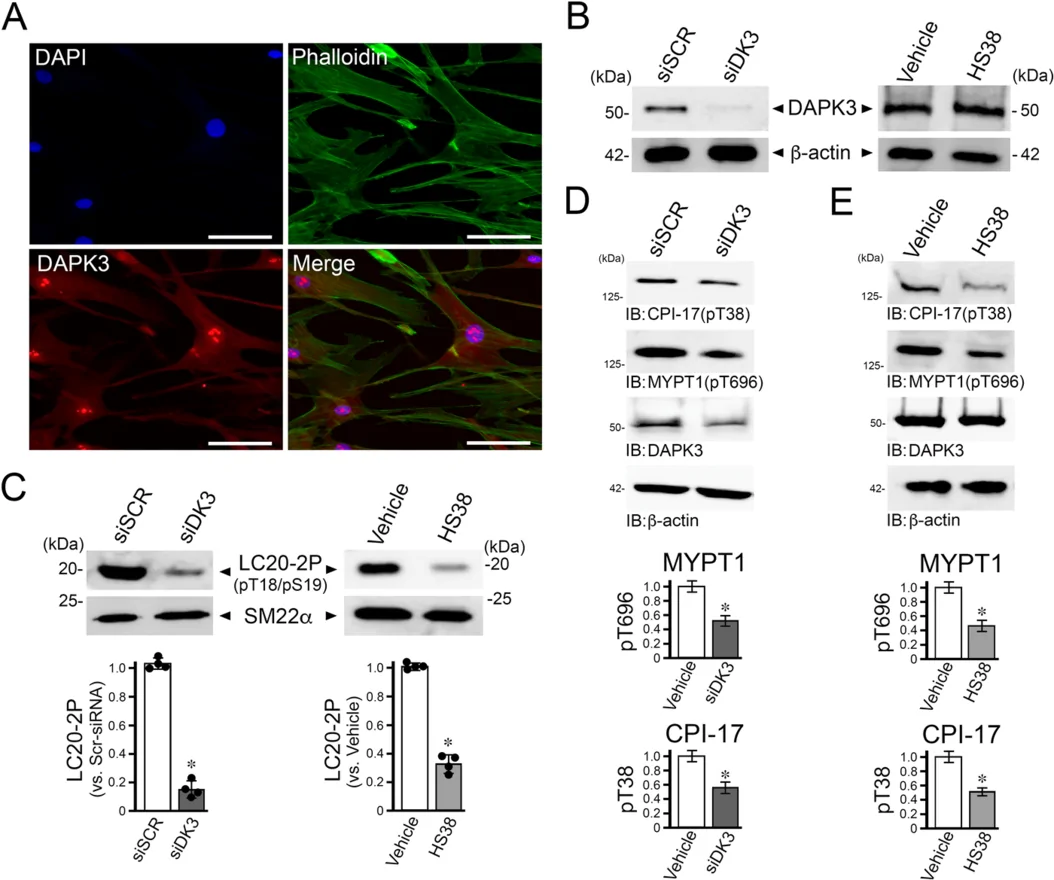
DAPK3 inhibition impacts LC20 phosphorylation in CASMCs. (A), Human coronary artery smooth muscle cells (CASMCs) were fixed and probed with AlexaFluor488‐phalloidin (green, F‐actin cytoskeleton), anti‐DAPK3 with AlexaFluor568‐conjugated secondary antibody (red), and DAPI nuclear stain (blue). Scale bars = 30 μm. (B–E), CASMCs were treated with: siRNA against DAPK3 (siDK3, 20 nM) or scrambled control siRNA (siSCR, 20 nM) for 24 h; and HS38 (25 μM) or vehicle (DMSO) control for 16 h. Whole cell lysates were probed by western blotting for (B) DAPK3 and (C) LC20‐pT18/pS19 diphosphorylation. Phosphorylation of myosin phosphatase target subunit (MYPT1‐pT696) and C‐kinase‐potentiated inhibitor protein (CPI‐17‐pT38) was also examined after DAPK3 silencing (siDK3, D) and inhibition (HS38, E). β‐actin or SM22α was used as a loading control. The statistical analyzes in C–E were performed using an unpaired Student's t‐test. All graphed values represent means ± S.E.M. for *n* = 3–4 independent experiments. Significantly different from the siSCR or vehicle (DMSO) control: *p < 0.05.

We previously reported that silencing of *DAPK3* with siDAPK or inhibition of DAPK3 activity with HS38 disrupted the F‐actin cytoskeleton organization in CASMCs. These changes in cytoskeletal arrangements were associated with reductions in FAK‐pY397 phosphorylation [19]. In this study, we confirm the impact of HS38 treatment on the phosphorylation of FAK‐pY397 in CASMCs with detection of concentration‐ and time‐dependent effects (Figure 2A,B respectively). In addition, immunocytochemical analysis revealed an HS38‐dependent attenuation in total signal intensity for FAK‐pY397 immunoreactivity in CASMCs (Figure 2C). Our findings also demonstrate that FAK‐pY576 phosphorylation was not altered by siDAPK3 or HS38 treatment of CASMCs (Figure 2D). The phosphorylation of Y576 in the activation loop of the kinase domain is dependent on the binding of Src family kinases to FAK after autophosphorylation of Y397. The phosphorylation of Y576 promotes maximal FAK activity and facilitates the activation of downstream pathways that regulate cell adhesion, migration and proliferation [2, 3]. So, the fact HS38 had no impact on FAK‐pY576 levels suggests the decrease in Y397 phosphorylation elicited by HS38 treatment could block subsequent FAK activation events.

**Figure 2:**
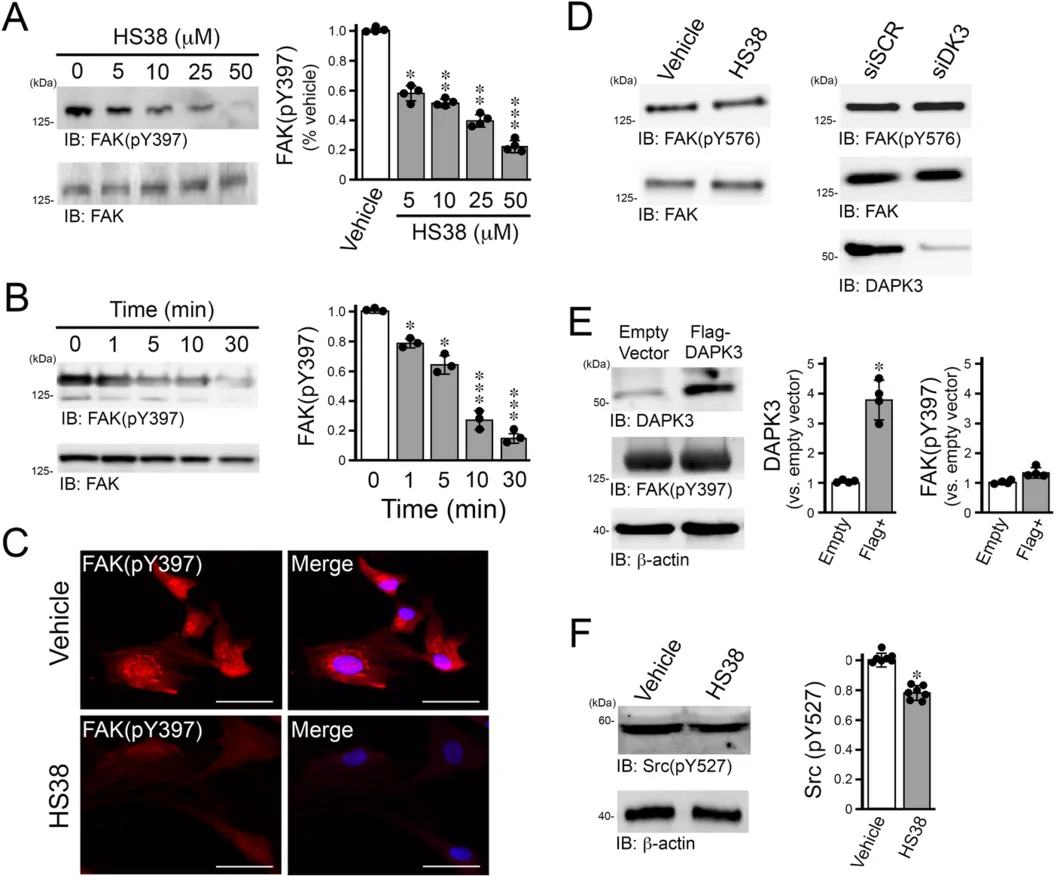
Suppression of DAPK3 signaling in CASMCs attenuates FAK‐pY397 phosphorylation. (A), CASMCs were treated with increasing [HS38] for 16 h, and FAK‐pY397 phosphorylation was quantified with normalization to total FAK signal. (B), The time‐dependent change in FAK‐pY397 phosphorylation was examined in CASMCs treated with HS38 (25 μM) for 0–30 min. FAK was used as loading control. (C), Immunocytochemistry was completed on CASMCs fixed and probed with anti‐[pY397]‐FAK and AlexaFluor568‐conjugated secondary antibodies (red). DAPI was used to stain the nuclei (blue). Scale bars = 30 μm. (D), FAK‐pY397 phosphorylation status was examined in CASMCs subjected to over‐expression of FLAG‐tagged DAPK3. (E), FAK‐pY576 phosphorylation was also examined following HS38 (25 μM, 16 h) or siDK3 (20 nM, 24 h) treatments; western blots are representative of *n* = 2 independent experiments. (F), Src activation status was probed with anti‐[pY527]‐Src after HS38 exposure (25 μM, 16 h), and phosphorylation was quantified with normalization against β‐actin as a loading control. Differences among [HS38] or timed treatments in A and B were analyzed by one‐way ANOVA with Dunnett's *post hoc* test. The statistical analyzes in *E* and *F* were completed using an unpaired, Student's *t*‐test. All quantified values represent means ± S.E.M; *n* = 3–4 independent experiments in each group; *p < 0.05; **p < 0.01; ***p < 0.001 versus controls.

We also found the ectopic over‐expression of Flag‐DAPK3 in CASMCs to have no effect on FAK‐pY397 levels (Figure 2E). This result would suggest that DAPK3 does not directly phosphorylate FAK at Y397. Conversely, Nehru and colleagues previously reported a decrease in FAK‐pY397 phosphorylation levels with over‐expression of DAPK3 in BJ/SV40T fibroblasts [18]. The unique cellular environments (i.e., fibroblasts vs. VSMCs) may account for the different findings.

FAK autophosphorylation on Y397 critically provides a high affinity binding site for the SH2 domain of Src. The phosphorylation of Src at Y527, by C‐terminal Src kinase (Csk), maintains the kinase in an inactive, autoinhibited conformation. The interaction of FAK and Src allow for the activation of the latter kinase since binding displaces the phosphorylated Y527 tail and relieves autoinhibition [8]. We found a small decrease in the level of Src‐pY527 following HS38 treatment of CASMCs (Figure 2F). This result suggests that HS38 treatment of CASMCs suppresses Src‐FAK complex formation with the majority of the Y397 site of FAK remaining unphosphorylated and the majority of Src remaining in an autoinhibited (pY527) conformation.

### 3.2 HS38 Treatment of CASMCs Leads to Nuclear Translocation of FAK

A substantive pool of FAK was found to shuttle to the nucleus of CASMCs with HS38 exposure, coincident with the dephosphorylation of the pY397 site. Following cell fractionation, FAK was mainly associated with the cytoplasmic pool under control (vehicle) conditions (Figure 3A), but the treatment of cells with HS38 elicited a marked accumulation of nuclear FAK. A corresponding increase in nuclear FAK immunoreactivity was also observed with confocal microscopy following HS38 treatment (Figure 3B). Although this is a novel report for DAPK3 influence on FAK cellular localization, the nuclear shuttling of dephosphorylated FAK is well described in the literature with links to a variety of cellular functions (reviewed in: [5], The nuclear translocation of FAK was detected in the hypertrophic myocardium of spontaneously hypertensive rats exhibiting heart failure, which is suggestive of a role for FAK in the regulation of mechanical transduction through modulation of gene transcription in cardiac myocytes [26]. Nuclear FAK was previously shown to modulate transcriptional programming of contractile genes in VSMCs [27, 28], the control of VSMC proliferation through transcriptional control of cyclin‐dependent kinase [29], the regulation of cell survival by promoting turnover of p53 (tumor suppressor) activity through the murine double minute‐2 (Mdm2)‐dependent p53 ubiquitination pathway [30], the downregulation of vascular cell adhesion molecule 1 (VCAM‐1) expression by stimulating GATA4 transcription factor ubiquitination [31], and the control of heterochromatin remodeling by the transcriptional induction of CpG‐binding protein 2 (MBD2) through activation of histone deacetylase 1 (HDAC1) dissociation [32]. Research also shows nuclear FAK can control chromatin accessibility at specific gene promoter and enhancer regions, altering transcriptional activity for genes tied to cell growth and proliferation [33].

**Figure 3:**
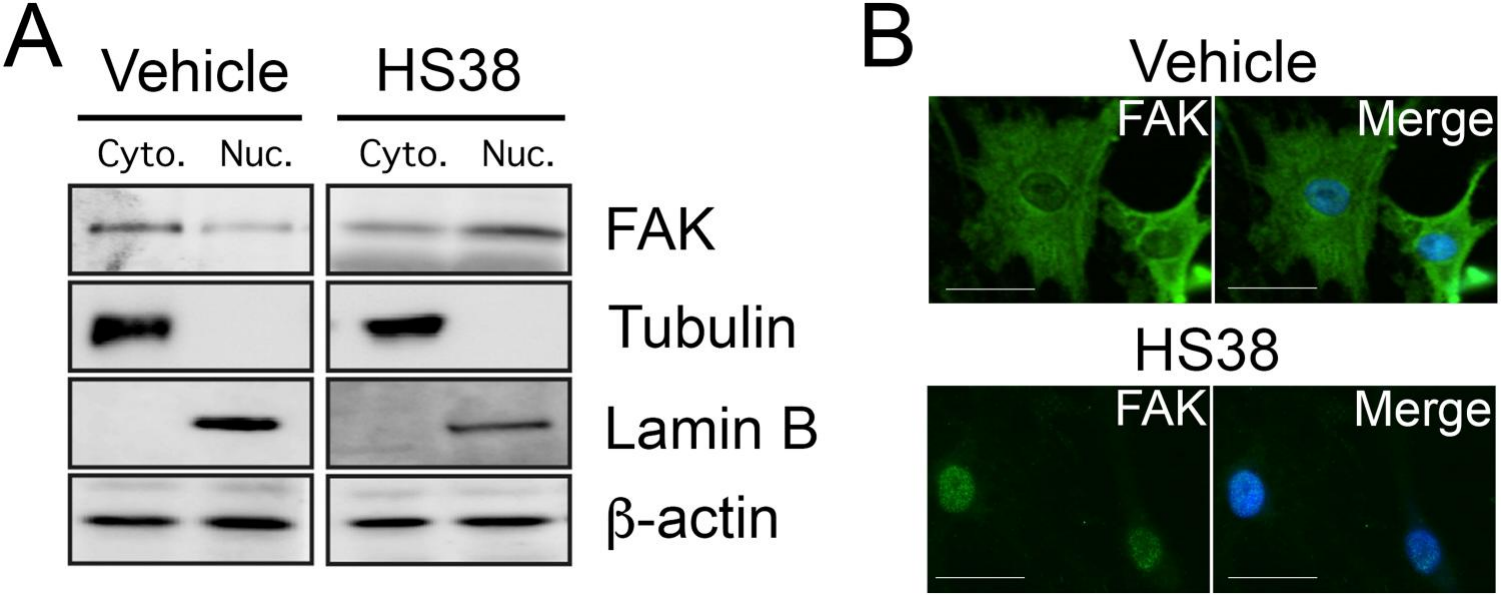
HS38 treatment of CAMSCs alters FAK subcellular localization. Coronary artery smooth muscle cells (CASMCs) were treated for 10 min in the absence (vehicle, DMSO) or presence of HS38 (25 μM) before analysis. (A), The nuclear localization of FAK was assessed following centrifugal fractionation of cell homogenates. Lamin B was used as a nuclear marker (Nuc.), and tubulin was used as a cytoplasmic marker (Cyto.). (B), Immunocytochemistry was completed on CASMCs fixed and probed with anti‐FAK, AlexaFluor488‐conjugated secondary antibodies (green) and DAPI nuclear stain (blue). Scale bars = 30 μm.

### 3.3 HS38 Treatment Suppresses the Activation State of Various Focal Adhesion Proteins

We completed further investigations of HS38(DAPK3)‐dependent events on various FAK‐targeted proteins in CASMCs. A well‐characterized target of Src‐mediated signaling downstream of FAK is the Crk‐associated substrate, p130Cas (also known as the breast cancer anti‐estrogen resistance protein, BCAR1). Y249 and Y410 are key phosphorylation sites of p130Cas linked to the motility and adhesion of cells. Once phosphorylated, these residues facilitate protein‐protein interactions between p130Cas and downstream signaling molecules, such as adapter proteins Crk and CrkL [34]. In our study, a significant decrease in p130Cas‐pY410 phosphorylation was detected in addition to a small, but non‐significant, decrease in p130Cas‐pY249 levels when CASMCs were exposed to HS38 (Figure 4A). The Y410 site is used as an effective proxy of p130Cas activation status. The phosphorylation of Y410 also serves as an indicator of Src involvement upstream of p130Cas [35] and is the dominant phosphorylation event recorded for p130Cas during the contractile activation of VSMCs [36]. Additionally, p130Cas signaling was shown to modulate the migration and proliferation of pulmonary VSMCs in pulmonary arterial hypertension [37].

**Figure 4:**
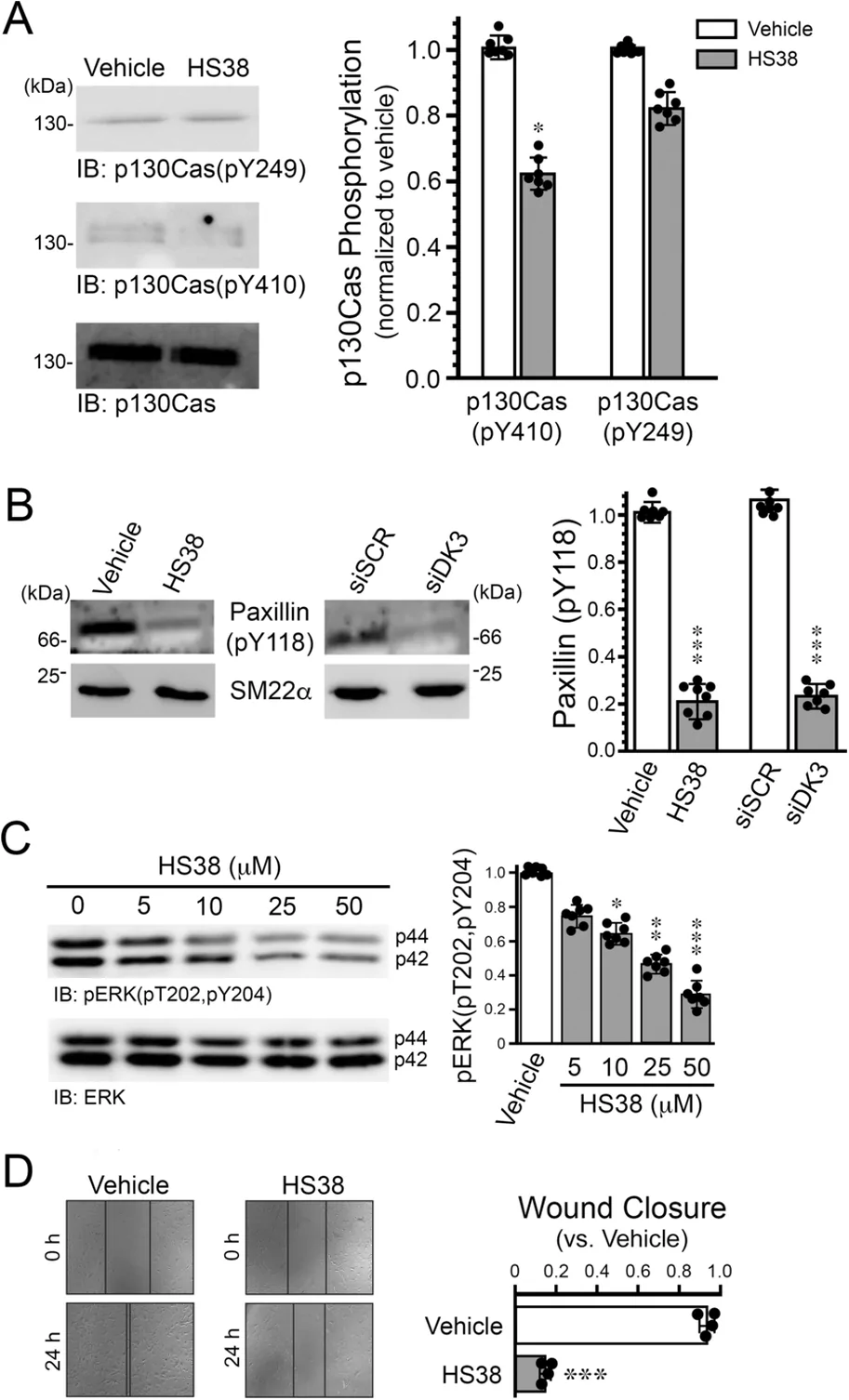
Suppression of DAPK3 signaling in CASMCs attenuates focal adhesion protein phosphorylation. (A), Proteins from whole cell lysates of CASMCs, incubated for 16 h with HS38 (25 μM) or DMSO (vehicle), were examined by western blotting and densitometric quantification following immunoreaction with anti‐[pY249]‐p130Cas and anti‐[pY410]‐p130Cas. p130Cas(E1L9H) was used as a loading control for quantification of p130Cas phosphorylation levels. (B), The phosphorylation of paxillin (pY118) following HS38 or siRNA treatment was quantified by western blotting with SM22α muscle protein used as loading control. (C), Phosphorylated ERK (pT202/pY204) was quantified by western blotting with normalization to total ERK protein (*n* = 7). (D), CASMCs were grown to 80% confluency, serum‐starved in the presence of Vehicle (DMSO) or HS38 (25 μM, 30 min) and then subjected to a cell migration assay. Cell migration was monitored and expressed as the % replacement of the initial cell‐free zone (*n* = 4). The statistical analyzes were performed using an unpaired, Student's *t*‐test (*p* < 0.05 vs. vehicle), except differences among HS38 treatments in *C* were analyzed by one‐way ANOVA with Dunnett's *post hoc* test (*n* = 7 independent experiments in each group; *p < 0.05; **p < 0.01; ***p < 0.001 vs. vehicle control). All graphed values represent means ± S.E.M.

The phosphorylation of paxillin at Y118 is also linked to the recruitment of the FAK‐Src signaling complex [Lopez‐Colome et al., 2017]. Pharmacological attenuation of paxillin‐pY118 phosphorylation (or mutation of the Y118 site) was previously shown to slow the disassembly of focal adhesions and suppress cell motility [10]. For this reason, we examined whether HS38‐treatment of CASMCs impacted paxillin‐associated signaling events. Coincident with the decrease in FAK activation (i.e., reduced pY397 levels, Figure 2), we observed a significant decrease in paxillin‐pY118 when CASMCs were treated with either siRNA‐DAPK3 or HS38 (Figure 4B). The activating phosphorylation of ERK (pT202/pY204) was also suppressed by the application of HS38 to CASMCs (Figure 4C). The localized activation of ERK2 at focal adhesions is facilitated by its association with phosphorylated paxillin‐pY118, and signaling to ERK2 can promote FAK release from existing focal contacts [10]. Finally, treatment with HS38 also elicited marked attenuation of CASMC migration (Figure 4D) in a scratch wound assay. There is abundant evidence that molecular interference of FAK inhibits the motility of numerous cell types, including VSMCs [5, 38]. In this regard, increased cell motility is associated with FAK‐pY397 phosphorylation and the downstream signals coordinated by the activated FAK‐Src complex.

## 4 Conclusion

Taken together, our experiments now reveal that treatment of CASMCs with HS38, a selective DAPK inhibitor generated by structure‐activity relationships of DAPK3, can elicit a collection of signaling events at the focal adhesion downstream of FAK. The autophosphorylation of FAK at Y397 is a key initiating event in focal‐adhesion signaling that promotes the time‐residency of FAK at focal adhesions which in turn leads to Src recruitment and the subsequent phosphorylation of downstream substrates [2, 3]. Thus, reduced Y397 phosphorylation would be expected to impair key focal‐adhesion signaling events and would disrupt adhesion and survival mechanisms, rather than necessarily preventing focal‐adhesion assembly itself. Importantly, a previous transcriptomic study of siRNA‐mediated ZIPK (DAPK3) knockdown in human arterial smooth muscle cells identified 390 differentially expressed genes (Deng et al., 2015). Notably, FAK (*PTK2*) was not among the genes differentially expressed following DAPK3/ZIPK knockdown, suggesting that the effects of DAPK3 inhibition on FAK signaling are unlikely to be mediated through its transcriptional regulation.

Notwithstanding the significant impact HS38 treatment has on signaling events associated with the focal adhesion complex, the precise signaling mechanism whereby DAPK3 acts on FAK remains undefined. It is highly unlikely that DAPK3 would directly phosphorylate FAK at either Y397 or Y576. There is no prior evidence to support DAPK3 phosphorylation of Tyr residues; therefore, the effect of HS38 on FAK‐pY397 phosphorylation is likely to be an indirect consequence of inhibiting DAPK3‐dependent phosphorylation of other substrates. Additionally, there are no direct links between DAPK3 and FAK identified in the literature, and past OMIC experiments that examined the off‐target effects of HS38 did not identify any inhibitory potential for HS38 against FAK or Src in activity screens of the kinome [23, 24]. Additional focused investigations will be needed to determine what is the key signal integrator that links DAPK3 with the FAK‐dependent regulation of focal adhesion events in VSMCs. In this context, three potential opportunities for signaling cross talk between DAPK3 and FAK are plausible.

First, Merlin (moesin‐ezrin‐radixin‐like member of the Band 4.1 family of cytoskeletal linker proteins) is a component of focal adhesion mechanosensing complexes and can modulate the activity state of FAK [39, 40]. Merlin is, itself, a phosphoprotein under the control of the PPP1R14A:PPP1C complex (i.e., myosin phosphatase, MLCP), and phosphorylated Merlin is required to maintain FAK activation (i.e., pY397 levels) [40]. As a proposed model, the HS38 treatment of CASMCs could relieve DAPK3 inhibitory signaling on MLCP; this action could in turn diminish Merlin phosphorylation and reduce its interactions with downstream partners within the focal adhesion environment. At its logical conclusion, HS38 treatment could provide titration of Merlin activity, via release of DAPK3 inhibitory phosphorylation events on PPPR14A (i.e., MYPT1‐pT696; Figure 1D), to attenuate the critical FAK‐pY397 phosphorylation that is necessary for focal adhesion disassembly and cellular motility.

Second, the human cell‐division cycle 14A (CDC14A) dual specificity phosphatase is co‐localized with F‐actin at the leading cell edges of migratory HCT116 (colorectal carcinoma) cells [41], and a direct interaction of CDC14A with DAPK3 was previously identified using yeast two‐hybrid screening, *in vitro* pull‐down assays, and co‐immunoprecipitation [42]. Although CDC14A was not detected at focal adhesions of HCT116, CDC14A knockout did provide an enhanced migratory phenotype with decreased focal adhesions and actin cytoskeleton remodeling. Thus, it is possible that HS38 effects could be transmitted from DAPK3 to FAK via CDC14A as an intermediary. It is tempting to speculate that CDC14A acts as a FAK phosphatase to target the pY397 site. However, phospho‐proteomic investigations by Chen and colleagues show CDC14A to act preferentially on pSer residues [43]; thus, it is unlikely that CDC14A directly targets FAK downstream of DAPK3.

Third, we presume the disruption of the actin cytoskeleton observed with HS38 treatment of CASMCs is accompanied by reduced intracellular tension. It is well known that changes in tension that originate from the actin cytoskeleton can influence the stability, size and composition of focal adhesions [44]. For example, cellular tension generated by physiological stretching was previously found to reveal cryptic vinculin binding sites on the talin mechanosensory protein that connects integrins to the actin cytoskeleton [45]. Cellular tension also enhanced the phosphorylation status of p130Cas [46] and caused conformational changes in the structure of FAK that promote its catalytic activation [47]. Past findings have also revealed DAPK3 to regulate epithelial cell behavior through cytoskeleton‐associated control of signaling events; in this case, DAPK3 inhibition influenced the Hippo pathway through non‐canonical signaling that involved cytoskeletal remodeling [48].

Most prominently, HS38 treatment is associated with a marked loss in the FAK autophosphorylation of its Y397 site. Thus, we would hypothesize that HS38 primarily impacts the molecular events that act upstream to enable this activation mechanism. The HS38 impact on FAK and downstream signaling in CASMCs mirror what is also observed with a specific small molecule inhibitor of FAK (FAK‐IN‐14; Supporting Information Figure S2) that acts primarily by blocking the autophosphorylation of the Y397 site.

FAK can act as a mechanosensing unit of the focal adhesion [15, 47]. In one scenario, cellular tension is thought to promote a direct change in FAK structure that enables the release of its catalytic activity and autophosphorylation potential. In another, paxillin is proposed to act as an adapter protein to connect the force transduction component vinculin with the induction of FAK activation [15]. When taken together, our results support a model wherein HS38 and DAPK3 control the tensile forces of VSMCs that are important for FAK activation. Presumably, HS38 acts to attenuate DAPK3‐dependent myosin phosphorylation (Figure 1C; Supporting Information Figure S3), which would in turn promote cytoskeletal disorganization and the loss of mechanical tension.

Although HS38 has inhibitory potential against DAPK1, its effective action on DAPK3 was substantiated using independent methods (i.e., silencing with siDAPK3). Regrettably, the potential off‐target impact of HS38 on DAPK1 is a limitation, yet no pharmacological agents exist to distinguish between DAPK3 and DAPK1 signaling. Although DAPK family members exhibit distinct cellular expression profiles and mechanisms of activation [17], future investigations could consider additional biochemical assessments with siRNA silencing of DAPK1 as a comparison.

## Author Contributions

All persons designated as authors qualify for authorship, and all those who qualify for authorship are listed. Abdulhameed Al‐Ghabkari conducted the experiments, completed data analysis, prepared figures and co‐wrote the manuscript. David A. Carlson synthesized the HS38 compound. Timothy A. J. Haystead coordinated the production of HS38 inhibitor and made intellectual contributions to the project. Justin A. MacDonald conceived and coordinated the study, assisted with experimental design, co‐wrote the article, provided trainee supervision, and made intellectual contributions to the project. All authors approved the final version of the article and agree to be accountable for all aspects of the work in ensuring that questions related to the accuracy or integrity of any part of the work are appropriately investigated and resolved.

## Supporting information


Supporting File

## Data Availability

The data that supports the findings of this study are available in the supporting information of this article.
