# Supplementary material for: Regulation of Focal Adhesion Kinase Signaling by Death‐Associated Protein Kinase 3 in Vascular Smooth Muscle Cells
Source: J Cell Biochem. 2026 Nov 15;127(11):e70139. doi: 10.1002/jcb.70139 (PMC13770492; doi:10.1002/jcb.70139)

Supplemental Figures

for

Regulation of focal adhesion kinase signaling by death-associated protein kinase 3 in vascular smooth muscle cells

Abdulhameed Al-Ghabkari[1], David A. Carlson[2], Timothy A. J. Haystead[2], and Justin A. MacDonald[1]

[1]Department of Biochemistry & Molecular Biology, Cumming School of Medicine, University of Calgary, Calgary, Alberta, T2N 4Z6, Canada

[2]Department of Pharmacology and Cancer Biology, Duke University Medical Center, Durham, North Carolina, 27710, USA

Supplemental Figure S1. Cell viability assessment of HS38-treated CASMCs. Cell viability was tested using the AlamarBlue assay (ThermoFisher). The data express the percentage of CASMCs that remained alive after exposure to HS38 (1 and 10 μM, 24 hours). A 96-well plate containing CASMCs in SmGM-2 media was incubated with vehicle (DMSO) or HS38; AlamarBlue stock solution was diluted 1:10 with fresh media and then added to the wells. After 4 hours, UV/VIS absorbance (570 nm) was used to determine cell viability as per the manufacturer's instructions.

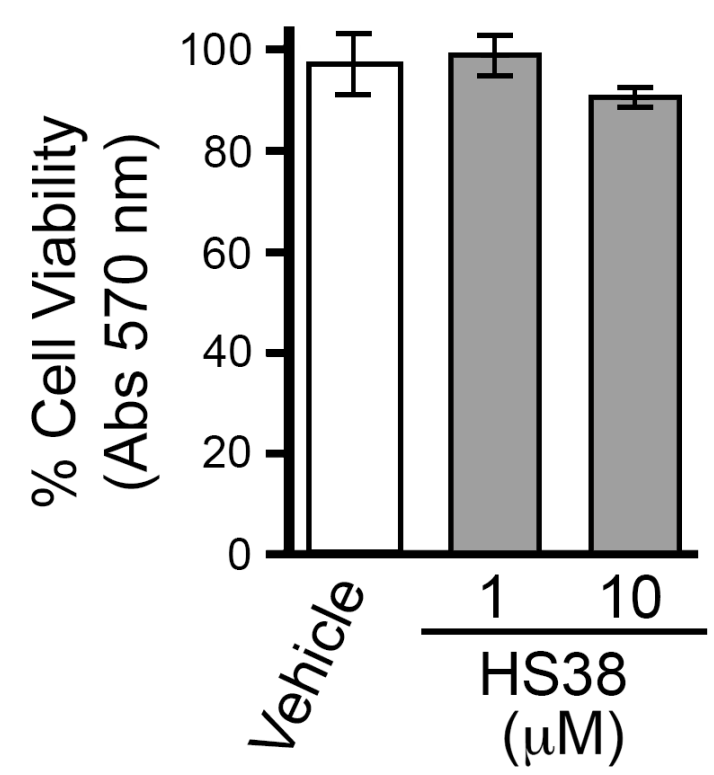

Supplemental Figure S2. Effect of FAK-IN-14 on FAK signaling pathways. Human coronary artery smooth muscle cells (CASMCs) were treated with the selective FAK-IN-14 inhibitor. In ***A***, FAK (pY397) and ERK1/2 (pT202,pY204) phosphorylation was examined in cells treated with FAK-IN-14 (25 and 50 µM, 24 hours). In ***B***, a scratch assay was used to assess CASMC migration. A scratch wound was made on confluent CASMCs, followed by treatment with either with Vehicle (DMSO) or FAK-IN-14 (50 µM, 24 hours). Cell migration was expressed as the percentage of cell replacement into the initial cell-free zone. In ***C***, CASMCs were fixed and probed with anti-FAK and goat anti-rabbit AlexaFluor568-conjugated secondary antibody to examine the localization of FAK (red) after Vehicle or FAK-IN-14 (50 µM) treatments. DAPI was used as a nuclear stain (blue). Scale bars = 30 µm. Values represent means ± S.E.M. for n = 4 independent experiments. Significantly different from the vehicle control (*A,* one-way ANOVA with Dunnett's post-hoc test, * - $p < 0.05$; *B*, Student's t test, *** - $p < 0.001$.

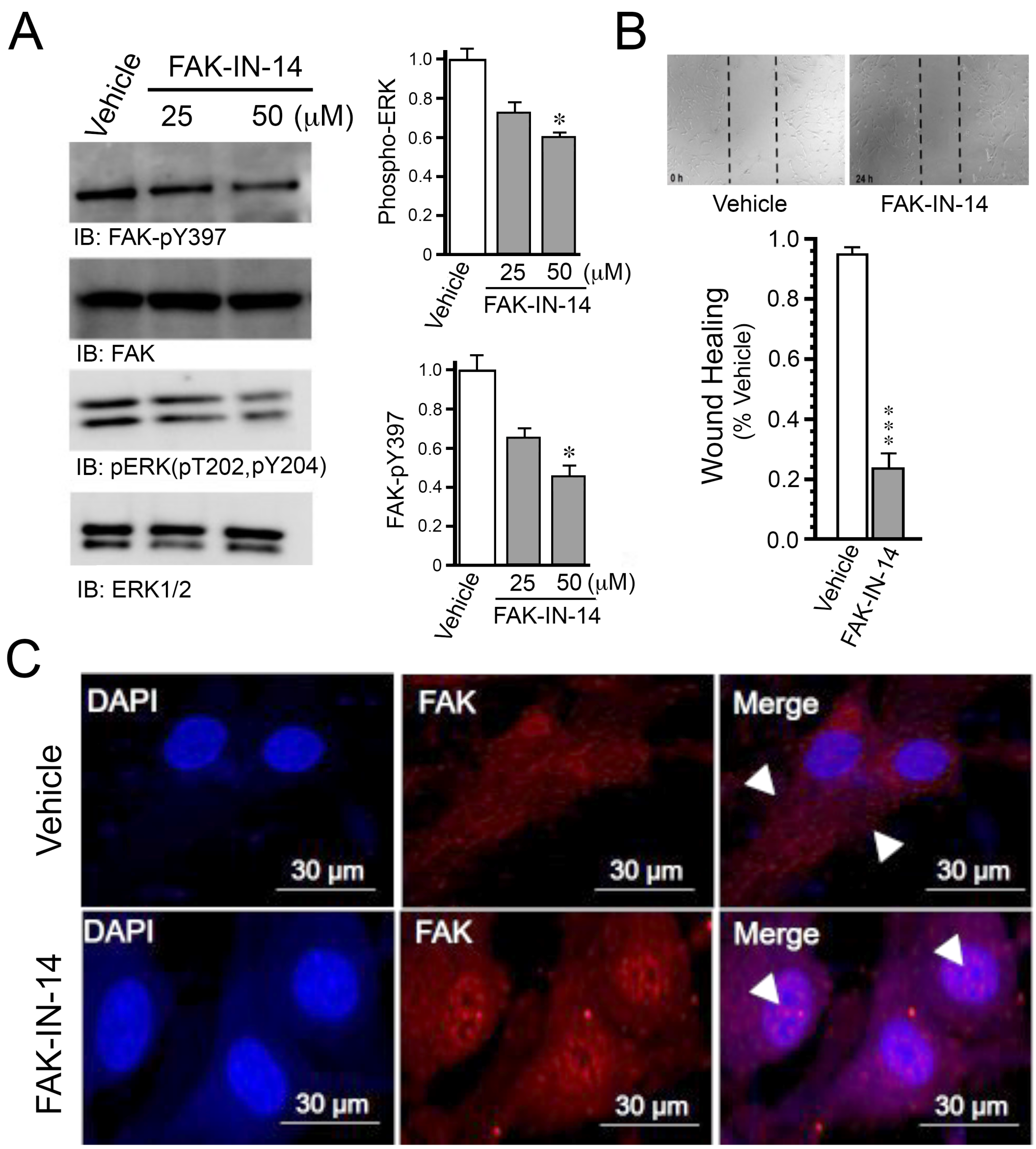

Supplemental Figure S3. HS38 treatment impacts LC20 phosphorylation in CASMCs. Human coronary artery smooth muscle cells (CASMCs) were incubated in the presence of vehicle (DMSO) or HS38 (25 μM, 24 hours). CASMCs were then fixed and probed with anti-LC20-pT18/pS19 and goat anti-rabbit AlexaFluor568-conjugated secondary antibody to examine LC20 phosphorylation (red). DAPI was used as the nuclear stain (blue). Scale bars = 30 μm.

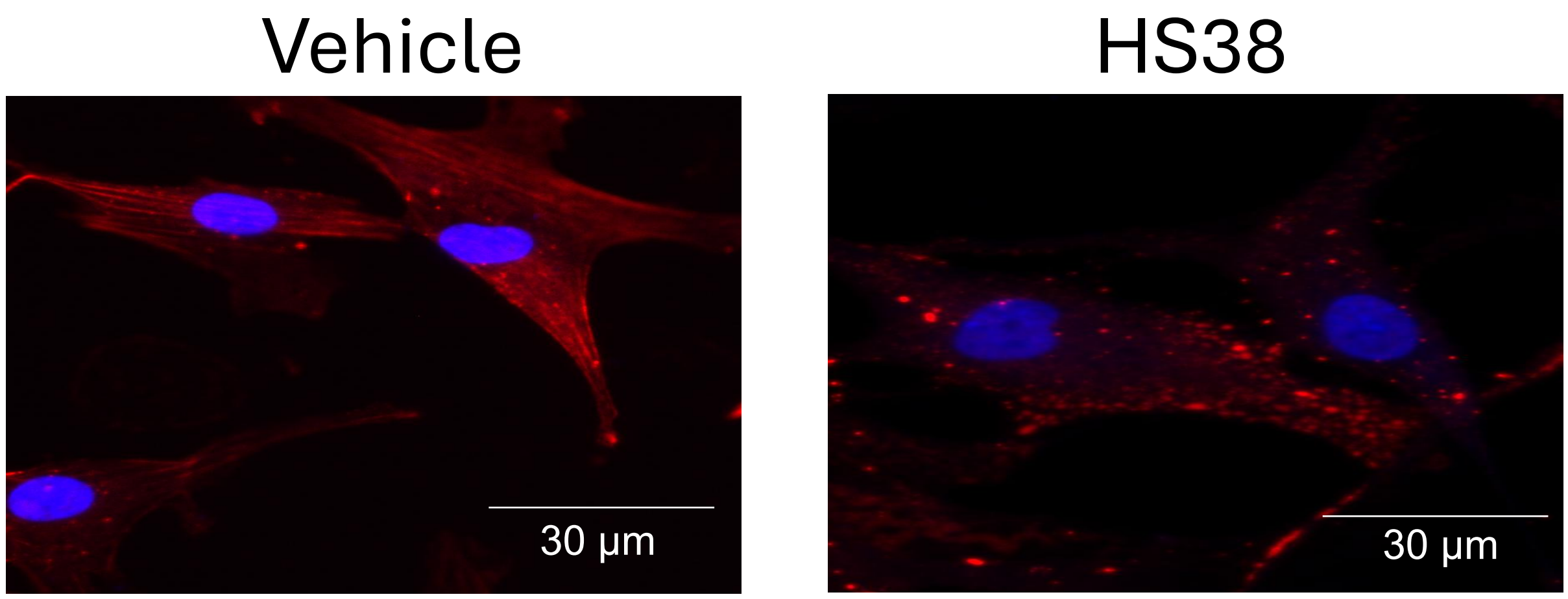

Supplement: Supplementary file 1 [file JCB-127-e70139-s001.pdf]
